# Surgical outcomes of the brachial plexus lesions caused by gunshot wounds in adults

**DOI:** 10.1186/1749-7221-4-11

**Published:** 2009-07-23

**Authors:** Halil Ibrahim Secer, Ilker Solmaz, Ihsan Anik, Yusuf Izci, Bulent Duz, Mehmet Kadri Daneyemez, Engin Gonul

**Affiliations:** 1 Department of Neurosurgery, Gulhane Military Medical Academy, 06018 Etlik-Ankara, Turkey; 2 Department of Neurosurgery, Kocaeli University Medical Faculty, Kocaeli, Turkey

## Abstract

**Background:**

The management of brachial plexus injuries due to gunshot wounds is a surgical challenge. Better surgical strategies based on clinical and electrophysiological patterns are needed. The aim of this study is to clarify the factors which may influence the surgical technique and outcome of the brachial plexus lesions caused by gunshot injuries.

**Methods:**

Two hundred and sixty five patients who had brachial plexus lesions caused by gunshot injuries were included in this study. All of them were male with a mean age of 22 years. Twenty-three patients were improved with conservative treatment while the others underwent surgical treatment. The patients were classified and managed according to the locations, clinical and electrophysiological findings, and coexisting lesions.

**Results:**

The wounding agent was shrapnel in 106 patients and bullet in 159 patients. Surgical procedures were performed from 6 weeks to 10 months after the injury. The majority of the lesions were repaired within 4 months were improved successfully. Good results were obtained in upper trunk and lateral cord lesions. The outcome was satisfactory if the nerve was intact and only compressed by fibrosis or the nerve was in-contunuity with neuroma or fibrosis.

**Conclusion:**

Appropriate surgical techniques help the recovery from the lesions, especially in patients with complete functional loss. Intraoperative nerve status and the type of surgery significantly affect the final clinical outcome of the patients.

## Background

Peripheral nerve injuries participate 10% of all injuries, and in 30% of extremity injuries [1]. Brachial plexus injury represents a severe, difficult-to-handle traumatic event. In recent years, the incidence of such injuries has gradually increased and the indications for surgery have been challenged. Most information on the results of brachial plexus repairs after missile injury has been derived from military reports. Brooks reported the first large series in 1954 [2], followed by a few other authors reported their series [3-11]. Studies regarding missile injuries of the peripheral nerves have shown that these injuries may be produced by low-velocity and high-velocity missiles that cause compressing and stretching of the nerves [7,12,13]. The high-velocity missile injuries are the second most common cause of brachial plexus lesions, accounting for about 25% [14].

Missile wounds, particularly those causing bone fractures, increased the risk of nerve severance and irreparable damage [15]. In addition, other extensive injuries like soft tissue; visceral organ and blood vessel injuries complicate the treatment and prognosis of the peripheral nerve injuries.

The patient's outcome depends on the characteristics and site of injury, the coexisting lesions, time of surgery, intraoperative findings, surgical technique, and postoperative physical rehabilitation. In this paper, we present our experience with 265 patients who had brachial plexus lesions caused by gunshot wounds.

## Methods

### Patient population

We reviewed the data of 265 patients with gunshot wounds who underwent evaluation and treatment for 288 brachial plexus lesions between 1966 and 2007 at the Department of Neurosurgery, Gulhane Military Medical Academy. Twenty-three patients were spontaneously recovered without surgery; most of them had minimal sensory deficits and partial lesions in electromyoneurography (EMNG) with lower trunk lesions. All patients who were treated surgically (242 patients) were men and the mean age was 22-years (ranging between 19 and 30 years). One hundred and six patients had shrapnel injury and 159 patients had bullet injury.

### Physical and Neurological evaluation

The physical examination usually began with inspection of the overall symmetry and observation of obvious scars related to either the initial trauma or subsequent surgery. The range of motion of all joints and the neck were assessed. The supraclavicular and infraclavicular areas were inspected and palpated for obvious scarring or bony spurs. Calluses from malunions of the clavicle can be palpated, and their presence could suggest compression of the underlying plexus.

It was important to keep in mind that high-velocity and fragmentary agents like grenades and land-mines frequently cause nerve injury at several levels. Manual muscle testing began by observing the muscle atrophy, the tone of each muscle group, and the muscle force. Examination of sensibility included deep pain, touch and pin sensation, two-point discrimination and some tactile location. A positive Tinel sign, elicited by tapping the supraclavicular area, was a strong indicator of nerve rupture. Damage to these nerves caused pain, numbness, and weakness in the shoulder, arm, and hand. The pain could be severe, and was often described as burning, pins and needles, or crushing. In general, the C5 nerve controls the rotator cuff muscles and shoulder function, C6 controls flexing the arm at the elbow, C7 partially controls the triceps and wrist flexion, and C8, T1 controls hand movements. When C5 and C6 are predominantly affected, the most common symptom is referred to as an Erb's palsy; these patients are unable to lift their arm or flex at the elbow, and severe atrophy can occur in the shoulder muscles. Another pattern of injury is when C8 and T1 are heavily damaged. These patients have hand weakness and pain, although some finger movement may remain. The most severe type of injury is when the arm is completely paralyzed as a result of extensive brachial plexus injury. All brachial plexus lesions underwent neurological evaluations in the preoperative stage and at the end of the follow-up period postoperatively. The muscle strength grading and, sensorial grading scales were used for the evaluation of outcome according to the preoperative time period, intraoperative nerve status, repair level, type of surgery, and length of the graft. Coexisting damage around the nerve lesion site were also listed. Because all patients were soldiers none of the data were lost in the follow-up period.

### Site of injury

The location of the lesions was defined according to the trunk, cord or nerve parts of the brachial plexus elements. Injuries were located in the supraclavicular region in 22 (8.3%) patients, and in the infraclavicular region in 243 (91.7%) patients. The number of nerve element injuries resulting from shrapnel wounds was higher than the number of injuries caused by missile wounds as documented in Table 1.

**Table 1:** Summary of the surgically treated brachial plexus lesions according to the injury site and wounding agent.

Location of Injury in Surgical Group	Number of Elements Evaluated Operatively		
	*Missile Injury*	*Shrapnel Injury*	*Total*

*Spinal nerve to trunk or trunk (supraclavicular)(n = 22)*			

C5–C6 to upper trunk or upper trunk	7	6	13
C7 to middle trunk or middle trunk	11	12	23
C8 to T1 to lower trunk or lower trunk	4	3	7

*Divisions to cord or cord (n = 141)(infraclavicular)*			

Lateral	24	43	67
Medial	82	87	169
Posterior	19	38	57

*Cord to nerve or nerve (n = 102) (infraclavicular)*			

Lateral to musculocutaneous	13	18	31
Lateral to median	17	13	30
Medial to median	15	29	44
Medial to ulnar	12	11	23
Posterior to radial	21	37	58
Posterior to axillary	10	9	19

Total (265)	235	306	541

### Initial surgical treatment

Soon after the injury, but before the nerve repair, all patients underwent initial surgical treatment of the gunshot wounds, especially for the shrapnel injuries. Plastic, vascular, chest and orthopedic surgeons repaired the soft tissue defects, blood vessels, hemothorax or pneumothorax, and bone fractures near the nerve. The coexisting lesions around the nerve injury site were detected during this initial evaluation, and the axillary and subclavian arteries were those most often affected. After the resection of necrotic soft tissues, the general or vascular surgeon performed reconstruction of the blood vessels if necessary. Seventeen bone fractures which were coexisted with nerve lesions were treated by orthopedic surgeons. The skin defects were treated by plastic surgeons immediately after injury, using skin flaps or epidermal skin grafts in 43 patients. 19 patients had the muscle defects including pectoralis major, pectoralis minor, deltoid muscle and sternocleidomastoid muscle. These muscles fragments disrupt the normal anatomy of the brachial plexus region, cause adhesions, and increase the risk of vascular and neural damage during the surgery. Most of these defects were caused by shrapnel injury which was secondary to landmine explosions. Hemothorax and/or pneumothorax was detected in 6 patients with brachial plexus lesions and treated by chest surgeons.

Most patients underwent initial management within the field military hospital without a neurosurgeon or with insufficient equipment to evaluate and to treat the nerve injury. After the initial procedures, the patients who were injured in other cities were transported to our department for peripheral nerve lesions. Nevertheless, when the initial surgeons found nerve transsection inside the wound and if the nerve defect was short and both nerve stumps were exposed, the surgeons had to approximate nerve stumps to each other with 1–2 paraneural nylon or silk sutures. If the gap was too long, they had to tack the accessible stumps down to the surrounding tissue.

### Timing of the repair

Indications for surgery included loss of nerve function without clinical and electrophysiological improvement in the early post-injury months. Surgical procedures were performed from 6 weeks to 10 months after injury. The majority of the lesions, 149 (56.23%) of 265, were repaired within the first 4 months. But early surgeries, during the first two months) were performed in a few of cases, who had total transected nerve elements that reported during the initial surgical procedures. Only 21 (7.92%) lesions were repaired between 8 and 10 months after injury because these lesions were followed-up by the orthopedic surgeons for bone fractures and wound infections before the operation for nerve lesion. As previously described in the section of *'initial surgical treatment*', most of the lesions were repaired within the first 6 months after injury. Incomplete functional loss and/or incomplete and limited functional recovery during the observation period were the reasons for delayed surgery. These patients were followed up monthly by clinical and electrophysiological examinations during the observation period.

### Intraoperative findings and surgical procedure

Operations were performed under general anesthesia. The patient was placed in opposition and incisions were made in the usual manner, except in cases of localized circumstances in the repair region (extensive scarring, skin flap, external skeletal fixation material, and severe contracture), which required some modifications. Microsurgical instruments and microscope were used especially during the decompression, neurolysis and anastomosis of the neural elements. The majority of the intraoperative findings (65.26%) were intact nerve elements, compressed by fibrosis, while 14 (2.58%) were completely ruptured nerve elements, 39 (7.21%) were nerve elements in which nerve continuity was interrupted by neuroma or fibrotic tissue at the stumps, 25 (4.62%) were partial nerve element rupture, and 110 (20.33%) were intact nerve elements surrounded by fibrosis.

Surgical procedures included end-to-end interfascicular anastomosis with sural nerve graft with or without neuroma excision (EEIA-SG) (4.44%), end-to-end epineural anastomosis with or without neuroma excision (EEEA) (7.95%), end-to-end interfascicular anastomosis with or without neuroma excision (EEIA) (9.05%), partial neuroma excision with EEIA-SG (PNE+EEIA-SG) (2.22%), partial neuroma excision with EEEA (PNE+EEEA) (3.51%), partial neuroma excision with EEIA (PNE+EEIA) (4.44%), interfascicular neurolysis (IN) (29.02%), exploration with simple decompression and external neurolysis (SD + EN) (39.37%). Intraoperative nerve stimulation techniques have been used to assess the nerve function in most cases since the early 1980s, but this was not systematically practiced. If the nerve was intact and compressed by the fibrosis, stimulation and recording electrodes were placed on the nerve. Direct intraoperative recording of nerve action potentials (NAP) guided management decisions; if action potential was transmitted across the lesion, external neurolysis alone was performed. Neurolysis was mostly accomplished both proximally and distally to the involved segment, and potential areas of entrapment were released. When the scar tissue could not be removed appropriately from the nerve, the epineurium was dissected and interfascicular neurolysis was performed. Simple external neurolysis was used in 353 lesions, and interfascicular neurolysis in 110 lesions.

Complete nerve rupture and interruption with the neuroma or fibrosis at the stumps were noted in 53 lesions. The stumps could be separated in some lesions still in the same plane, and the stumps in the others, were directed to different planes, sometimes grabbed by adjacent callus or abundant scar tissue. If the structures such as fibrosis were seen without response to nerve stimulation, after the dissection of the epineurium, these fibrotic parts of the nerve were removed. If there were fascicles-in-continuity, and intact electrophysiologically, we protected them and performed decompression on these nerve fibers. End-to-end epineural or interfascicular anastomoses were performed at the nerve defect due to excision of fibrotic parts of the nerve. In 55 lesions, we performed partial neuroma excision and end-to-end epineural or interfascicular anastomosis with or without using sural nerve grafts.

Proximal and distal nerve stumps and non-transmitting nerve segments were resected until the appearance of normal fascicles and vascular architecture with healthy epineurium. The non-transmitting segments were characterized by abnormal color, unusual consistency, and/or sparse or absent vascularization. Sometimes they were soft or, conversely, diffusely fibrotic in cases when long-term local infection existed near the nerve. The nerve defect was repaired by an end-to-end epineural anastomosis in 62 lesions, end-to-end interfascicular anastomosis in 73 lesions, and end-to-end interfascicular anastomosis with sural nerve graft in 36 lesions, by using monofilament interrupted silk or nylon suture (Ethilon 8-0; Ethicon, Inc, Somerville, NJ). Before the choice of suturing technique, the nerve stumps were mobilized reasonably, without tension at the suture sites and the risk of wound dehiscence and, if it was possible, anastomosis was performed without nerve grafting. Otherwise, repair with a nerve graft was necessary. We used interfascicular technique (two or four grafts) and the sural nerve was preferred as nerve graft. This nerve graft divided into two or four sections and end-to-end anastomosed to the nerves using interfascicular technique. The length of the nerve gap was measured after resection, and maximum mobilization of the nerve stumps and graft was about 10% longer than the corresponding nerve defect. Physical therapy was applied soon after injury in some cases, as well as after surgery in all cases. We did not use the nerve transfers or neurotization as a surgical method.

### Effects of coexisting injuries in the repair region

Gunshot-related damage on the soft tissues, vascular structures, bones, muscular structures, and visceral organs, was frequently noted in the repair region; in our series, coexisting injuries were detected in 95 of the 265 cases; bone fractures in 17, big vascular injuries in 10, skin defects in 43, muscular defects in 19, and hemothorax/pneumothorax in 5 cases. Most of the tissue and muscular defects were caused by shrapnel wounds. Statistical analysis was performed on the relationship between the final outcome and the injury level, the timing of repair, the intraoperative nerve status, the type of surgery and the length of sural nerve graft, using a chi-square test. The statistical significance was based on the p < 0.05 level.

## Results

After the mean postoperative follow-up period of 20 months (range between 6 and 39 months), the motor and sensory recovery were scored on a scale ranging from 0 to 5 points, as recommended by the British Medical Research Council [16]. The sensory recovery scale was slightly modified, as seen in Table 2. A large number of the lesions were ≤S2 and M2 levels before the operation. The results were classified into three groups. Good outcome was defined as ≥M4 and ≥S4, fair outcome was represented by M2–M3/S2–S3, and poor outcome was ≤M1 and ≤S1. Twenty-three patients (7.98%) who had minimal motor and sensorial deficits spontaneously recovered.

**Table 2:** Modified British Medical Research Council (BMRC) grading of sensorimotor recovery, and motor recovery on the quality of outcome after brachial plexus repair [16].

*Motor recovery*		
Poor	M0	No contraction
	M1	Return of perceptible contraction in the proximal muscles
Fair	M2	Return of perceptible contraction in both proximal and distal muscles
	M3	Return of perceptible contraction in both proximal and distal muscles of such of degree that all important muscles are sufficiently powerful to act against resistance
Good	M4	Return of function as in stage 3 with the addition that all synergic and independent movements are possible
	M5	Complete recovery

Sensory recovery		

Poor	S0	No sensation
	S1	Deep pain re-established
Fair	S2	Some response to touch and pin, with over-response
	S3	Good response to touch and pin, without over-response
Good	S4	Location and some tactile discrimination
	S5	Complete recovery

### Pain Management in Brachial Plexus Injuries

Injury to the brachial plexus may cause severe pain. Intractable pain was assigned in 5 cases in our series with lower trunk lesions. Three of them exposed shrapnel injury and the others exposed missile injuries. Pain usually starts a few days after the initial trauma and can be intractable. It is commonly described as continuous, burning, and compressing and is frequently located in the hand. All the patients were initially treated with carbamazepin, amitriptyline, gabapentin, some antidepressants and sympatholytic agents, and antipsychotic drugs. Excision of the neuroma and reconstruction of the nerve was also the best treatment of the pain. In our patients, the early exploration and reconstruction of the brachial plexus not only improved the function of the arm but also relieved the pain.

### Final clinical outcome and prognostic factors

#### Surgical level

Although the majority of the repairs had fair results, the good results were achieved in upper trunks (53.85%) and lateral cords repairs (40.30%). The poor results were significantly high in lower trunks (28.57%), medial cords (21.89%), and ulnar nerves (21.74%). (Table 3) The results were not statistically significant because the p values were 0.268 when comparing spinal nerves and trunks, 0.074 when comparing the divisions and cords and 0.851 when comparing the cords and nerves.

**Table 3:** Relationship between the final outcome of the brachial plexus lesions which were treated surgically and the location of the lesion.

Final Outcome for Repair Level (%)			
Location of Injury in Surgical Group	*Good*	*Fair*	*Poor*

*Spinal nerve to trunk or trunk*			

C5–C6 to upper trunk or upper trunk	53,85	38,46	7,69
C7 to middle trunk or middle trunk	30,43	60,87	8,7
C8 to T1 to lower trunk or lower trunk	14,29	57,14	28,57

*Divisions to cord or cord*			

Lateral	40,3	50,75	8,96
Medial	26,63	51,48	21,89
Posterior	38,6	49,12	12,28

*Cord to nerve or nerve*			

Lateral to musculocutaneous	29,03	58,06	12,9
Lateral to median	36,67	56,67	6,67
Medial to median	31,82	59,09	9,09
Medial to ulnar	21,74	56,52	21,74
Posterior to radial	32,76	58,62	8,62
Posterior to axillary	21,05	63,16	15,79

#### Time of operation

When we evaluated the results according to sensory and muscle strength grading, good outcome was achieved in the first 4 months (44.97%). The rate of the good outcomes decreased when the preoperative interval was increased; good outcome was noted in only 14.29% of the lesions in which the operation was delayed more than 8 months. We could not get enough useful recoveries at the time of surgery more than 8 months after injury. According to these results, the first 4 months after the injury seems to be the critical period for surgery; (Table 4) however, the result was not statistically significant, according to the chi square test (p = 0.129).

**Table 4:** Relationship between the preoperative time period and the final outcome.

The final outcome for preopertaive interval (%)				
	*0–4 months (n = 149)*	*4–6 months (n = 60)*	*6–8 months (n = 35)*	*8–10 months (n = 21)*

Poor	8,72	11,67	14,29	19,05
Fair	46,31	50	57,14	66,67
Good	44,97	38,33	28,57	14,29

#### Intraoperative findings and operative techniques

Significant good results were seen in lesions with nerve intact and only compressed by fibrosis (71.67%), and with neuroma and/or fibrosis in-continuity (52.08%). (Table 5) The majority of the results were fair in lesions with complete rupture (71.43%), interrupted by a neuroma and/or fibrosis at the end of the nerve (71.79%), and partial rupture (64.00%). These results were statistically significant (p < 0.05). Nine surgical techniques were performed in repairing the lesions, and the best outcome was found in the 54.93% of lesions in which the exploration with simple decompression and external neurolysis technique was used. Based on the surgical techniques, good recovery rates were 16.67% for EEIA-SG, 25.58% for EEEA, 30.61% for EEIA, 16.67% for PNE+EEIA SG, 26.32% for PNE+EEEA, 30.33% for PNE+EEIA, 49.68% for IN, and 54.93% for SD+EN. The majority of the results based on the surgical techniques were fair, with the exception of the exploration with simple decompression, external neurolysis, and interfascicular neurolysis. (Table 6) This results were statistically significant (p < 0.05).

**Table 5:** Relationship between the intraoperative nerve status and the final outcome.

The final outcome for intraoperative findings (%)					
	*Complete rupture (n = 14)*	*Interrupted by a neuromaor/and fibrosis at the stump (n = 39)*	*Partial rupture (n = 25)*	*Neuroma or/andfibrosis is continuity (n = 110)*	*Nerve is intact, only compressed by fibrosis (n = 353)*

Poor	21,43	20,51	16	5,45	3,68
Fair	71,43	71,79	64	43,64	24,65
Good	7,14	7,69	20	50,91	71,67

The final outcome for intraoperative findings (%)

**Table 6:** Relationship between the type of surgery and the final outcome.

The final outcome for type of surgery (%)								
	*EEIA-SG (n = 24)*	*EEEA (n = 43)*	*EEIA (n = 49)*	*PNE+ EEIA-SG (n = 12)*	*PNE+ EEEA (n = 19)*	*PNE+ EEIA (n = 24)*	*IN (n = 157)*	*SD+EN (n = 213)*

Poor	29,17	18,6	12,24	25	10,53	4,17	2,55	2,35
Fair	54,17	55,81	57,14	58,33	63,16	65,5	47,77	42,72
Good	16,67	25,58	30,61	16,67	26,32	30,33	49,68	54,93

#### Length of the graft

We used 3 cm grafts in 11 lesions, 3,1–5 cm grafts in 14 lesions, and 5.1 cm grafts in 11 lesions. The maximum length of the sural nerve graft was 6,5 cm. Good outcome was noted in 36.36% of lesions with grafts 3 cm or shorter, and in 14.29% of lesions in the 3,1 to 5 cm group. We did not get good results in the repairs with grafts more than 5,1 cm. Thus, 3 cm seems to be the critical length of the nerve graft to get good clinical outcome. (Table 7) However, the p value was 0.055 for the comparison of the relationship between the length of the graft and the final outcome, and the difference was not statistically significant.

**Table 7:** Relationship between the length of the graft and the final outcome.

The final outcome for the length of the graft (%)			
	*0–3 cm (n = 11)*	*3,1–5 cm (n = 14)*	*>5,1 cm (n = 11)*

Poor	0	35,71	45,45
Fair	63,64	50	54,55
Good	36,36	14,29	0

#### Complications

Ninety-five coexisting lesions in the nerve injury site were detected during the initial evaluation. Ten of these were vascular injuries that mostly affected the axillary and brachial arteries. In one case, the axillary artery was lacerated at the proximal repair line with the graft, during the dissection of the nerve elements, and the vascular surgeons repaired the artery. Two patients with land-mine wounds, developed osteomyelitis; we performed a simple decompression and external neurolysis technique in two nerve elements in one case, and interfascicular neurolysis in one nerve element in the other. After a course of antibiotics, and hyperbaric oxygen therapy for a month, these cases improved, and we did not propose additional surgery.

## Discussion

Brachial plexus lesions represent approximately 11.5% of our nerve injury population at the Gulhane Military Medical Academy. These lesions are technically difficult to explore and to treat; the anatomy is complex, great vessels are close to the plexus, and intraoperative vascular injury is a risk factor for surgery. As a consequence, we aimed to evaluate the final clinical outcomes and to determine the prognostic factors in patients undergoing surgical treatment for brachial plexus lesions resulting from gunshot wounds.

Although there have been some developments in microsurgical techniques, intraoperative neurophysiology, and new repair techniques, the surgical treatment of peripheral nerve injuries, resulting from gunshot wounds has not changed in its essentials since World War II [17]. The results of the gunshot wounds to the peripheral nerves are neuropraxia, axonotmesis, and/or neurotmesis injuries [18]. In older military series, low-velocity missiles, usually shell fragments that damaged by direct impact, caused the most of the injuries. These injuries involved neuropraxia or axonotmesis [10]. Patients with low-velocity missile injuries may display a significant return of function within a few months [19-21]. On the other hand, high velocity missiles (especially footman rifle) injuries have three mechanisms: direct impact, shock waves, and cavitation effects. These last two mechanisms are more common and cause nerve stretching and compression. Patient with high-velocity missile injuries have generally failed to display a significant return of function [10]. Although complete transsections were more common in missile injuries, there was no significant difference between shrapnel injury and missile injuries [22]. In the present study, most of the injuries were neurotmesis as a result of high-velocity missile injuries. Most of the patients with injuries of upper trunk and posterior cord with partial neurologic deficits, may display spontaneous neurological recovery, but not those with injuries of the lower elements [2,9]. In the published series, various numbers of cases with incomplete functional loss display a significant return of function [2,7,9]. In our series, only 23 patients (7.98%) who had minimal motor, and sensorial deficits were spontaneously recovered. The indication for surgery was the neurological deficit in the distribution of one or more elements of the plexus, without improvement between 6 weeks and four months after the injury. The injury affected one nerve element in 94 cases (87 of them exposed missile injury, and the others exposed shrapnel injury), two nerve elements in 74 cases (59 from missile injury, 15 from shrapnel injury), three nerve elements in 56 cases (9 from missile injury, 47 from shrapnel injury), and four nerve elements in 29 cases who exposed shrapnel injuries. Some authors have reported that the best results were obtained with an early operation and repair of the nerve injuries [9]. If lesion-in continuity was found with neurological examination and electrophysiological tests, resection was delayed for 3 to 6 months to allow for possible spontaneous recovery. When there was no of spontaneous recovery during this period, resection of the lesion was indicated.

The time of the surgery for nerve injuries was largely dependent on patients' referral, which may cause a significant delay. The nerve must be surgically explored within 3 months after injury, if no significant functional recovery is noted [23-25]. Surgery delayed up to 6 months was not pragmatically unfavorable during this period, surgery was indicated if anatomic recovery seemed to stop or fail, if there were differences between the motor and sensorial recoveries, or if there was uneven functional recovery with regular chronology but an absence of improvement in some muscles [4]. If surgery is delayed longer than 1 year, results will not be good, and this may be one of the reasons for conservative treatment [4,8].

Generally, the clean wound without infection, a stable fracture, restoration of circulation and skin closure over neurovascular structures are priorities and should be reasons for delayed nerve repair [26]. Early surgical exploration is not indicated, because of the possibility of spontaneous recovery, and it is difficult to evaluate the extent and severity of the nerve damage [27]. This is one of the reasons for surgical delay in our series. Soon after the injury and before the nerve repair, all patients underwent initial surgical treatment of the missile wound, especially in cases with shrapnel injury. After they recovered without complications from the initial operation, they were admitted to us for definitive treatment of nerve lesions. The postoperative recovery period was a major reason for the surgical delay in this study because of the need for an observational period for spontaneous recovery. We performed surgical treatment in 209 cases within the first 6 months after injury.

According to some authors, the surgery on of brachial plexus lesions resulting from gunshot wounds was rarely profitable and justifiable because recovery at infraclavicular levels occurred better than that at supraclavicular levels [2,6]. In supraclavicular levels, the recovery at C5, C6, and some C7 spinal nerve repairs was better than that at C8, and T1 spinal nerve repairs. Neurolysis and surgical repair of the lower elements rarely improved functional recovery but only helped with pain relief. At the cord level, the results of repair were favorable for lateral and posterior cord and their outflows. In our series, we noted the best recovery results in upper trunk repairs, and suggesting that the adult patients with C8, T1 spinal nerves, lower trunk or medial cord incomplete lesions are suited for conservative treatment unless pain is not manageable by pharmacological means, because surgical repair have a low yield regarding ultimate functional recovery.

Studies regarding peripheral nerve injury caused by gunshot wounds have shown that most lesions are caused by both direct bullet trauma and by the indirect heat and shock to adjacent tissue [7]. These injuries present a specific problem in peripheral nerve surgery because of the mechanism of injuries. Gunshot wounds to the brachial plexus usually result in lesions- in- continuity, but, the patients with a large majority of these lesions-in-continuity had complete functional loss [2,6,7,11]. Intraoperative stimulation and NAP recording studies are important in assigning whether the nerve elements need resection or not. In our series, 13 nerve elements ruptured completely, and 38 elements were interrupted by neuroma or fibrosis. In 23 nerve elements, partial rupture was noted. The majority of nerve lesions-in-continuity were compressed by fibrosis in the present study. More than 50% of repaired nerves-in-continuity with neuroma or/and fibrosis and compressed by fibrosis had good outcome. The worst outcome was seen in lesions with completely ruptured nerve elements. Surgical procedure was determined with the operation microscope images and intraoperative stimulation and NAP recording studies. If the nerve is intact and has compressed or is surrounded by fibrosis and has partial ruptured nerve elements, the best way to evaluate the lesion of the nerve is to stimulate and record the nerve across the injury site by intraoperative nerve conduction stimulation. The presence or absence of an intraoperative NAP helps to determine further operative management. The presence of a NAP beyond an injury site indicates preserved axonal function or significant axonal regeneration, which augurs well for clinical recovery. The absence of a NAP has been correlated histologically with a Grade IV Sunderland lesion, inadequate regeneration and poor clinical recovery. NAP studies have been performed with all lesions in continuity [28-30]. The presence of NAP indicates neurolysis, and absence indicates that recovery will not proceed without resection and repair of the lesions [28-30]. The peripheral nerve has to be able to adapt to neurolysis and repair by slacking down (approximately 15% of their total length) and by elongation (4.5%) [31]. Except in patients treated with external and interfascicular neurolysis, the nerve stumps were mobilized before suturing so that no tension was exerted on the suture sites. If possible, anastomosis was performed without using nerve grafts. In some cases, repair with autograft was necessary. The length of the gap between nerve stumps was measured after resection, and maximal mobilization of the nerve stumps and graft was about 10% longer than the corresponding nerve defect. Useful functional recovery (Grade 3) was reported in more than 90% of neurolyzed cases [6,7,10]. In our series, good results were seen in 54.93% of the simple decompression and external neurolysis group, and in 49.68% of the interfascicular neurolysis group. According to Kline, approximately 69% of lesions repaired by suture and 54% of lesions repaired by grafts had successful outcomes [7]. In another study, the rate of recovery was 67% for primary suture, and 54% for nerve grafting [6]. Samardzic stated that the rate of the functional recovery was 87.8% among the lesions which were repaired by nerve grafts [10]. In our series, good results were obtained in 30.61% of end-to-end interfascicular anastomosis group, 30.33% of the partial neuroma excision and performed interfascicular anastomosis group, in 26.32% of the partial neuroma excision and performed epineural anastomosis group, and in 25.58% of the end-to-end epineural anastomosis group. The good results were achieved as the same ratio (16.67%) for the lesions repaired by partial neuroma excision and interfascicular anastomosis with sural nerve graft, and the interfascicular anastomosis with sural nerve graft with total neuroma excision or not.

Functional recovery after graft placement depends on the severity of injury and the graft length [11,17,23,24]. In addition, the small-caliber grafts are better than larger-caliber grafts [32,33]. We used sural nerve grafts, which are small-caliber nerves. Although many authors have stated that the length of the nerve defect influences outcome, experimental data have revealed that other factors may also contribute to the poor results after the use of long nerve grafts [34]. Good results are possible in cases of long nerve defects [35], although, along with numerous other authors [11,23,24,36,37]. we found that worse results correlated with increased graft length. We obtained good outcome in 36.36% of lesions repaired with 3 cm or shorter sural nerve graft and suggest that 3 cm is the critical length of the nerve graft to get good functional outcome.

A few studies address the dependence of nerve repair outcomes on comorbidities fractures, main vascular lesions, and soft tissue (skin, muscle) defects and so on in the nerve repair region [6,11,24,38]. These comorbidities may influence the final outcome of the nerve in its own manner: for example great vascular lesions aggravate the results through ischemia, and bone fragments may cause additional nerve damage during the initial missile trauma or subsequent callusing spreads around the repaired nerve. An associated vascular injury will warrant emergency repair [39,40]. In addition to transections of a major vessel, gunshot wounds involving the brachial plexus may produce pseudoaneurysms or arteriovenous fistulas that compress the plexus and produce progressive loss of function and severe pain. Injured elements need to be dissected and gently moved away from the area of vascular repair. In our series, there were coexisting injuries in 95 of the 265 cases.

## Conclusion

Since peripheral nerve injury has no fatal course but a spectrum of morbidity, appropriate repair of injured nerves is important in retaining quality of life for the patient. Although gunshot wounds usually leave the nerves intact, and several authors have stated that these lesions sometimes recover spontaneously; surgery is indicated for most of them. We conclude that appropriate surgical techniques help recovery, especially in the lesions with complete functional loss. Intraoperative appearance of the nerve and the type of surgery are the prognostic factors of the patients' final functional outcome.

## Abbreviations

EMNG: Electromyoneurography; EEIA+SG: End-to-end interfascicular anastomosis with sural nerve graft; EEEA: End-to-end epineural anastomosis; EEIA: End-to-end interfascicular anastomosis; PNE+1: Partial neuroma excision with EEIA-SG; PNE+2: Partial neuroma excision with EEEA; PNE+3: Partial neuroma excision with EEIA; IN: Interfascicular neurolysis; SD+EN: Simple decompression and external neurolysis; NAP: Nerve action potentials.

## Competing interests

The authors declare that they have no competing interests.

## Authors' contributions

HIS designed the study, performed surgeries for many of these patients and drafted the manuscript. IS acquired the data. IA analysed the data and performed the statistical analyses. YI performed linguistic and technical corrections. BD made substantial contributions to conception and design of the study. MKD participated in the study design, performed surgeries for many of these patients and revised the manuscript. EG read and approved the final version of this manuscript. All authors read and approved the final manuscript.
